# Covid-19: knowledge, risk perception, trust and vaccination readiness among German medical students

**DOI:** 10.1192/j.eurpsy.2022.1358

**Published:** 2022-09-01

**Authors:** F. Baessler

**Affiliations:** Centre for Psychosocial Medicine, Department Of General Internal And Psychosomatic Medicine, Heidelberg, Germany

**Keywords:** Interdisciplinary teaching, medical education, vaccination, Covid-19

## Abstract

**Introduction:**

The Covid-19 pandemic has highlighted the urgency for innovative vaccine strategies since the best of vaccines cannot be useful if people do not accept vaccinations. The current situation suggests that vaccinology has been ignored in the medical curriculum and needs more representation in teaching.

**Objectives:**

What, where and how vaccinology is taught during medical studies in Heidelberg and development of an interdisciplinary revised syllabus and practice-oriented teaching methods.

**Methods:**

Curricular mapping of courses on the topic of “vaccination”, defining new learning objectives for designing innovative teaching units in consultation with teachers and students, redevelopment and updating of teaching materials.

**Results:**

In preliminary work, an OSCE has been created by students. Initial findings on the status of teaching on vaccinations and related communication skills in medical schools of Germany with respect to student needs and the national guidelines on learning goals for future physicians are submitted in journal ‘Vaccine’. In collaboration with the elective track Digital Medicine, two tele-OSCEs, an online knowledge quiz on vaccination education and a corresponding evaluation tool will be developed in WS 2021/22. Students will learn how to create modern teaching methods and evaluate them scientifically, using a concrete and relevant topic as an example, and will gain an insight into teaching.

**Conclusions:**

The findings will be integrated into the Heidelberg medical school curriculum (HeiCuMed) on a long-term basis by developing a comprehensive interdisciplinary module ‘Vaccination and Vaccines’, which can either be integrated into various existing courses (e.g. virology, medical Communication, global health, etc) or as a separate elective interprofessional course.

**Disclosure:**

No significant relationships.

